# Social Dysfunction in ADHD Children Associated With Increased Amygdala Functional Connectivity Instability: A Static and Dynamic Functional Connectivity Study

**DOI:** 10.1002/cns.71088

**Published:** 2026-08-23

**Authors:** Kangfuxi Zhang, Xuyao Pei, Jing Yuan, Yilu Zhao, Zhao Fu, Hang Yang, Xiaoyu Xu, Zaixu Cui, Qingjiu Cao

**Affiliations:** ^1^ NHC Key Laboratory of Mental Health (Peking University), Peking University Institute of Mental Health, Peking University Sixth Hospital Beijing Key Laboratory for Big Data Innovative Application of Child and Adolescent Mental Disorders, National Clinical Research Center for Mental Disorders (Peking University Sixth Hospital) Beijing China; ^2^ Shanxi Academy of Medical Sciences, Shanxi Bethune Hospital, Tongji Shanxi Hospital Third Hospital of Shanxi Medical University Taiyuan China; ^3^ Tongji Medical College, Tongji Hospital Huazhong University of Science and Technology Wuhan China; ^4^ Sichuan Provincial Center for Mental Health, School of Medicine, Key Laboratory of Psychosomatic Medicine, Chinese Academy of Medical Sciences, Sichuan Provincial People's Hospital University of Electronic Science and Technology of China Chengdu China; ^5^ Affiliated Mental Health Center & Hangzhou Seventh People's Hospital Zhejiang University School of Medicine Hangzhou Zhejiang China; ^6^ Chinese Institute for Brain Research Beijing China

**Keywords:** amygdala, attention‐deficit/hyperactivity disorder, dynamic functional connectivity, social dysfunction, static functional connectivity

## Abstract

**Aims:**

Social dysfunction (SD) is highly prevalent in children with attention‐deficit/hyperactivity disorder (ADHD), yet the underlying neural mechanisms remain poorly understood. The amygdala is a central hub for socio‐emotional processing, and altered functional connectivity (FC) may explain the heterogeneity of social dysfunction in ADHD.

**Methods:**

We examined both static FC (sFC) and dynamic FC (dFC) of amygdala subregions in 53 drug‐naïve ADHD with SD (ADHD+SD), 71 drug‐naïve ADHD without SD (ADHD–SD), and 72 typically developing controls (TDC). The sFC was computed by voxel‐wise correlations, whereas dFC variability was quantified using sliding‐window analysis. Group differences were tested with ANCOVA, and group‐dependent cluster–symptom associations were examined using interaction models.

**Results:**

No significant group differences were found for sFC. In contrast, dFC variability between the left basolateral amygdala (BLA) and the middle occipital gyrus (MOG) as well as the superior frontal gyrus (SFG) was significantly elevated in ADHD+SD relative to both ADHD−SD and controls, following the gradient ADHD+SD > TDC > ADHD−SD. Interaction analyses revealed a significant group‐dependent association between BLA–MOG variability and social motivation, whereas no significant group‐dependent associations were observed with core ADHD symptoms.

**Conclusions:**

These findings suggest that social dysfunction in drug‐naive children with ADHD is associated with abnormal dynamic, rather than static, amygdala connectivity. The ADHD+SD subgroup showed increased temporal variability in BLA‐centered circuits involving both visual‐perceptual (MOG) and executive control (SFG) systems. Furthermore, the group‐dependent association between BLA–MOG variability and social motivation suggests that altered amygdala‐visual temporal coordination may contribute to heterogeneity in social functioning. These results highlight dynamic amygdala‐cortical connectivity as a promising neural feature for characterizing clinically relevant social dysfunction in ADHD.

## Graphical abstract

We identified abnormal dynamic rather than static amygdala connectivity as a neural signature of social dysfunction in ADHD. In summary, the ADHD+SD subgroup exhibited the most pronounced amygdala network instability, and ADHD–SD children exhibited brain‐behavior coupling patterns resembling TDC, highlighting that temporal instability of amygdala networks, rather than static connectivity, differentiates clinically relevant social impairments in ADHD.

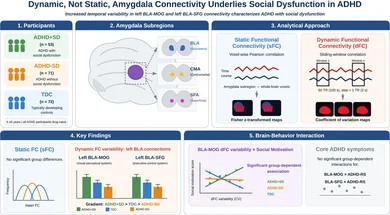

**Abbreviations**
ADHD: attention‐deficit/hyperactivity disorder
ADHD‐RS‐IV: ADHD Rating Scale‐IV
ADHD–SD: ADHD without social dysfunction
ADHD+SD: ADHD with social dysfunction
ASD: autism spectrum disorder
BLA: basolateral amygdala
BOLD: blood oxygen level‐dependent
CMA: centromedial amygdala
CV: coefficient of variation
C‐WISC III: Chinese Wechsler Intelligence Scale for Children, third version
DBS: deep brain stimulation
dFC: dynamic functional connectivity
EPI: echo planar imaging
FA: flip angle
FC: functional connectivity
FD: frame‐wise displacement
FOV: field of view
GRF: Gaussian random field
K‐SADS‐PL‐C DSM‐5: kiddie‐schedule for affective disorders and schizophrenia—present and lifetime version DSM‐5
LSD: least significant difference
MNI: Montreal Neurological Institute
MOG: middle occipital gyrus
SD: social dysfunction
SFA: superficial amygdala
sFC: static functional connectivity
SFG: superior frontal gyrus
SRS‐1: first edition of the Social Responsiveness Scale
TDC: typically developing controls
TE: echo time
TI: inversion time
TMS: transcranial magnetic stimulation
TR: repetition time

## 1 Introduction

Attention Deficit Hyperactivity Disorder (ADHD) is a neurodevelopmental condition characterized by inattention, hyperactivity, and impulsivity, significantly affecting children's daily functioning and social interactions [1]. Among children diagnosed with ADHD, an estimated 52%–82% also experience social dysfunction (SD), which further complicates their social and emotional development [2, 3]. These challenges often manifest as difficulties in forming and maintaining friendships, interpreting social cues, and engaging in appropriate social behaviors [4]. Notably, impairments in social functioning are more persistent and disabling than the core symptoms of ADHD and are associated with poorer long‐term outcomes [5]. Research indicates that ADHD's core symptoms alone cannot fully explain the observed deficits in social functioning, suggesting that ADHD with and without SD may involve distinct neural mechanisms [6]. However, few studies have examined the neural mechanisms differentiating ADHD with and without SD.

Recent systematic reviews have synthesized neuroimaging and electrophysiological evidence to explore the neural bases of social dysfunction in ADHD, focusing on the amygdala's role in social functioning [7]. The amygdala, a key brain region for social cognition and emotion regulation, plays a central role in the social brain [8]. However, the amygdala is not a homogeneous structure but consists of subregions, each with specialized functions and distinct connectivity patterns with other brain regions [9]. For example, the basolateral amygdala (BLA) is primarily connected to temporal and frontal regions and serves as the main receiver of sensory information [10]. The centromedial amygdala (CMA), which has a high density of oxytocin receptors, is linked to fear responses and social anxiety [11]. The superficial amygdala (SFA) modulates approach‐avoidance behaviors and processes socially relevant and reward‐related information [12, 13]. MRI studies have shown that individuals with ADHD exhibit altered amygdala volume and functional activity, which may contribute to their heightened sensitivity to external stimuli and increased impulsivity [7, 14, 15]. However, there is a significant gap in research exploring the distinct contributions of these subregions to social dysfunction in ADHD.

Functional connectivity (FC) reflects the relationships between neural activities in different brain regions and is crucial for understanding brain function integration [16], especially in neurodevelopmental disorders like ADHD [17]. Increasing evidence shows that interregional coupling fluctuates over time, including at rest, reflecting intrinsic dynamics that support flexible cognitive and affective processing [18]. Static FC (sFC) captures the overall, time‐averaged organization of brain networks, offering insights into stable, trait‐like aspects of long‐term brain organization [19]. In contrast, dynamic FC (dFC) captures the temporal variability of resting‐state FC, providing complementary insights into the capacity for adaptive reconfiguration and transient brain coordination [20, 21]. Combining sFC and dFC thus allows a more comprehensive characterization of both the stable and flexible components of neural communication. This integrated approach is particularly relevant for ADHD, where atypical fluctuations in network dynamics may underlie clinical heterogeneity.

Building on this framework, we focused on the amygdala as the seed region because of its central role in socio‐emotional integration and its dense reciprocal connections with the prefrontal, temporal, and occipital cortices [22, 23]. These widespread pathways support the coordination between social and cognitive control processes that are often disrupted in ADHD [24, 25]. Beyond its structural embedding, the amygdala displays intrinsic oscillatory activity across multiple frequency bands, which is temporally coupled with cortical networks involved in attention and social information regulation [26, 27]. These time‐varying properties suggest that dynamic analyses can more effectively capture subtle disturbances in amygdala coordination than static measures alone [20, 28, 29]. Examining both sFC and dFC therefore enables a joint assessment of stable and flexible features of amygdala‐based circuits, providing a framework to probe the neural basis of social dysfunction in ADHD.

Guided by this rationale, we investigated whether abnormal amygdala connectivity contributes to social dysfunction in ADHD by analyzing sFC and dFC patterns of amygdala subregions and the whole brain. Using the first edition of the Social Responsiveness Scale (SRS‐1), we classified participants with ADHD into two subgroups, those with social dysfunction (ADHD+SD) and those without (ADHD–SD), and compared both groups with typically developing controls (TDC). We hypothesized that ADHD+SD individuals would exhibit decreased sFC between the amygdala and other brain regions, as well as increased instability in dFC, compared to ADHD–SD and TDC.

## 2 Materials and Methods

### 2.1 Participants

A total of 136 medication‐naive ADHD participants were recruited from the outpatient department of Peking University Sixth Hospital. All participants were newly diagnosed and had never received pharmacological treatment for ADHD before. In addition, 81 age‐matched TDC were recruited from schools or nearby communities via recruitment advertisements. All patients were interviewed by experienced psychiatrists, meeting the diagnostic criteria for ADHD based on clinical interviews that follow the Diagnostic and Statistical Manual of Mental Disorders, Fifth Edition [1]. The Chinese version of the kiddie‐schedule for affective disorders and schizophrenia—present and lifetime version DSM‐5 (K‐SADS‐PL‐C DSM‐5) was used to interview the participants and their parents [30, 31]. The inclusion criteria for the ADHD group were as follows: (1) diagnosis of ADHD according to the K‐SADS‐PL‐C DSM‐5; (2) aged 6–16 years; (3) Chinese Wechsler Intelligence Scale for Children, third version (C‐WISC III) IQ ≥ 70; (4) were right‐handed and (5) had never received any psychotropic medication. Exclusion criteria included: (1) common ADHD comorbidities (autism spectrum disorder, oppositional defiant disorder, tic disorders); (2) any current or past psychiatric disorder other than ADHD; and (3) serious physical or neurological disorders or a history of brain injury. TDC participants met the same criteria but had no K‐SADS‐PL‐C DSM‐5 identified mental disorders.

This study was approved by the Medical Research Ethics Committee of the Peking University Sixth Hospital. All children agreed to participate. Participants aged ≥ 8 years provided written assent, and a parent or legal guardian provided written informed consent.

### 2.2 Clinical Measurements

The severity of symptoms was evaluated by the ADHD Rating Scale‐IV (ADHD‐RS‐IV) [32] and SRS‐1 [33, 34] by the participants' parent. The SRS‐1 is a widely used questionnaire designed to evaluate the degree of difficulties an individual experiences in social function. It is extensively applied in both research and clinical settings to identify and assess autism spectrum disorder (ASD) and other social function‐related disorders. The SRS‐1 includes five primary dimensions: social awareness (the ability to recognize social cues and environmental information), social cognition (the ability to understand and interpret the behavior and emotions of others), social communication (the ability to effectively communicate and interact with others), social motivation (the willingness to engage in and maintain social relationships), and autistic mannerisms (behaviors characteristic of ASD). Notably, higher SRS‐1 scores indicate more severe social impairment. To explore the social function in ADHD, we further subclassified participants in the ADHD group into two groups, ADHD+SD and ADHD−SD, according to the SRS‐1 *T*‐scores. Participants with *T*‐scores ≥ 60 were considered to have social dysfunction in accordance with previous Chinese studies [35].

### 2.3 MRI Data Acquisition

All MRI images were acquired on a GE Discovery 3.0T MR750 system at the Center for Neuroimaging in Peking University Sixth Hospital. Participants were instructed to relax with their eyes closed, stay still, and not think about anything systematically without falling asleep during the scans. The parameters of the T1 image were as follows: repetition time (TR) = 6.7 ms; echo time (TE) = 2.9 ms; inversion time (TI) = 450 ms; flip angle (FA) = 8; field of view (FOV) = 256 × 256 mm^2^; matrix = 256 × 256; slice thickness = 1.0 mm with no gap, 180 sagittal slices. Echo Planar Imaging (EPI) sequence was used to collect resting‐state functional images, parameters: 240 echo planar imaging volumes; 43‐slice axial scan (sequence is odd‐numbered slices first, then even‐numbered slices), slice thickness = 3.2 mm, slice gap = 0 mm; TR = 2000 ms, TE = 30 ms, flip angle = 90°, imaging matrix = 64 × 64, FOV = 220 × 220 mm^2^; and the imaging plane being parallel to the anterior commissure‐posterior commissure image plane.

### 2.4 MRI Data Pre‐Processing

We processed resting‐state functional images using RESTplus [36] on the MATLAB R2018a platform. Preprocessing included removal of the first 10 volumes, slice timing correction, realignment, coregistration of the functional images to each participant's structural T1 image, segmentation of the structural image into gray matter, white matter, and cerebrospinal fluid, and spatial normalization to the Montreal Neurological Institute (MNI) space using the DARTEL algorithm implemented in SPM12 (resampled voxel size = 3 × 3 × 3 mm^3^). The normalized images were then linearly detrended, nuisance covariates (Friston 24 motion parameters, white matter signal, and cerebrospinal fluid signal) were regressed out, and the time series were temporally band‐pass filtered (0.01–0.10 Hz).

Head motion was indexed by the mean frame‐wise displacement (FD) derived using Jenkinson's relative root mean square algorithm [37]. Participants with excessive head motion with > 3 mm of translation or > 3° of rotation in any direction or a mean FD exceeding 0.5 mm were excluded from further analysis.

### 2.5 Voxel‐Wise Static and Dynamic FC of Amygdala Subregions

The geometry and anatomical coordinate of each amygdala subregion are labeled according to the template determined by the stereotaxic probabilistic maps and implemented in FSL's Juelich histological atlas [9]. These regions were divided into the bilateral BLA, CMA, and SFA as mentioned above.

The sFC was defined as the Pearson correlation coefficient between the mean blood oxygen level‐dependent (BOLD) time series of each amygdala subregion and that of other voxels in the whole brain. The correlation coefficients were then converted to *z* values using Fisher's *z* transformation to improve statistical normality. Before the statistical analyses, the result maps were smoothed out with a 4‐mm full‐width at the half‐maximum Gaussian kernel.

**FIGURE 1:**
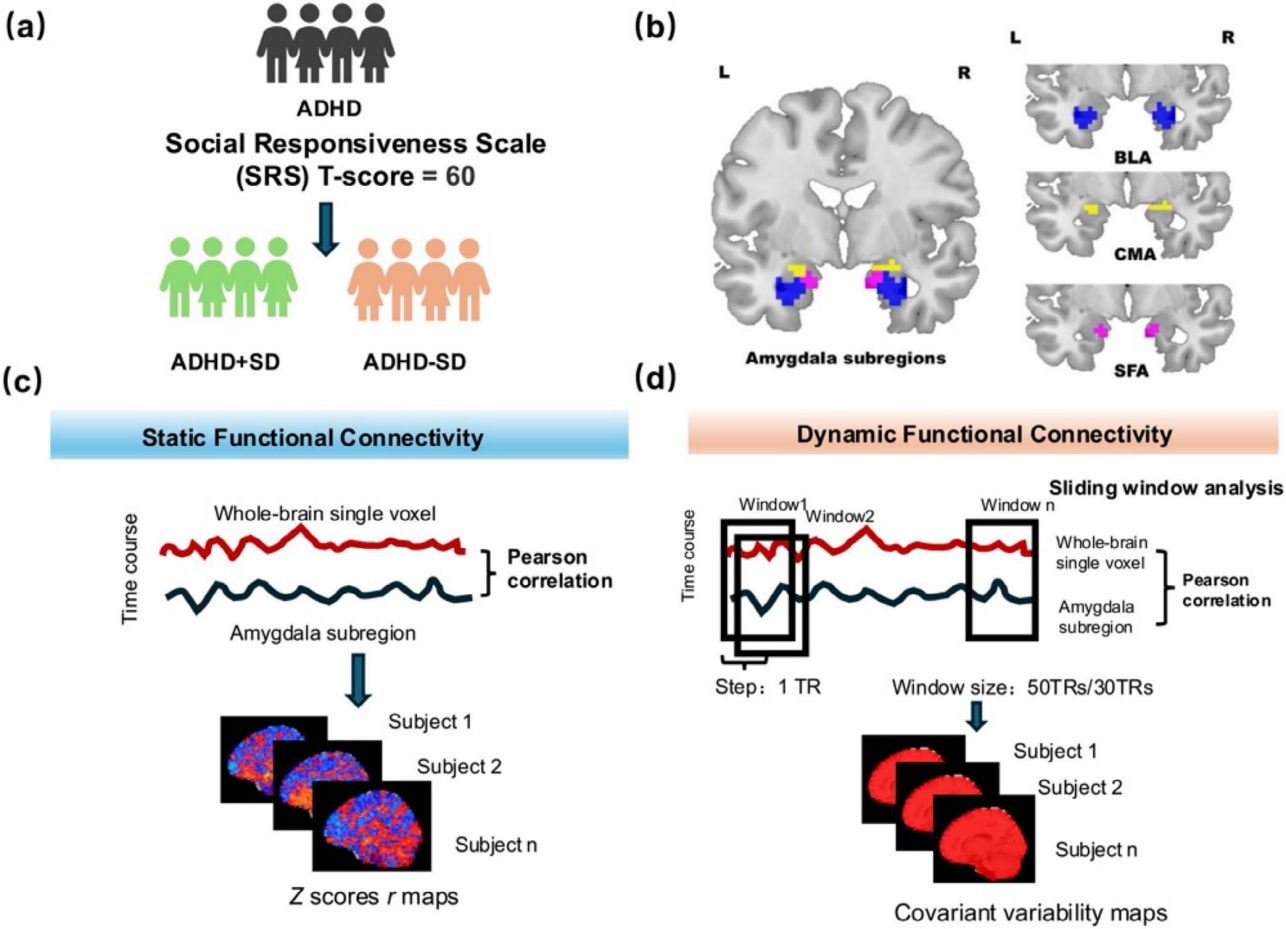
(a) Overview of the methodological pipeline. ADHD participants were stratified into subgroups with (ADHD+SD) or without social dysfunction (ADHD–SD) using the Social Responsiveness Scale (SRS; 1st ed.) *T*‐score cutoff ≥ 60. (b) The amygdala was parcellated into three subregions—basolateral (BLA), centromedial (CMA), and superficial (SFA). (c) Static functional connectivity (sFC) was computed as the Pearson correlation between the mean BOLD time series of each amygdala subregion and all other brain voxels (correlations Fisher *z*‐transformed before group statistics). (d) Dynamic functional connectivity (dFC) was estimated using a sliding‐window approach and summarized as temporal variability (e.g., coefficient of variation) across windows. ADHD, attention‐deficit/hyperactivity disorder; BLA, basolateral amygdala; BOLD, blood‐oxygen‐level‐dependent; CMA, centromedial amygdala; dFC, dynamic functional connectivity; SD, social dysfunction; SFA, superficial amygdala; sFC, static functional connectivity; SRS, Social Responsiveness Scale.

The dFC was estimated using a sliding‐window approach. A tapered Hamming window with a length of 50 TRs (100 s) and a step size of 1 TR (2 s) was employed, following previous methodological studies demonstrating that this parameter combination provides a reasonable balance between the temporal sensitivity required to capture dynamic fluctuations in FC and the statistical reliability of connectivity estimation [28, 29, 38]. Within each sliding window, Fisher's *z*‐transformed dFC maps were generated using the same procedure as the sFC analysis. The coefficient of variation (CV) across all sliding windows was then calculated for each voxel to quantify dFC variability. To evaluate the robustness of our findings, additional validation analyses were performed using alternative window lengths of 30 TRs, 40 TRs, and 60 TRs, as well as an alternative step size of 2 TRs. The above analysis process is shown in Figure 1.

### 2.6 Statistical Analyses

Demographic and clinical data were analyzed using SPSS 27.0. For normally distributed variables with homogeneous variance, one‐way ANOVA was performed, with post hoc comparisons conducted using the Bonferroni test. A chi‐squared test was used to compare the distribution of categorical data across groups. The significance threshold was set at *p* < 0.05.

Voxel‐based comparisons of both sFC and dFC maps were performed using one‐way ANCOVA, followed by post hoc analysis with the least significant difference (LSD) *t*‐test in the DPABI toolbox [39]. To correct for multiple comparisons, Gaussian Random Field (GRF) theory was applied (voxel‐level *p* < 0.005, cluster‐level *p* < 0.05). Confounding variables, including age, gender, IQ, and mean FD, were regressed out in all analyses. Only clusters with more than 50 voxels were considered significant due to the robustness of voxel‐level analysis [40].

To examine the clinical relevance of the significant clusters identified in the group‐level imaging analyses, linear interaction models were used to test whether the associations between abnormal brain regions and clinical symptom dimensions differed across groups. For each model, the clinical symptom scores (ADHD‐RS‐IV and SRS‐1 scores) were entered as the dependent variable, and the mean FC value or dFC CV value extracted from each significant cluster, group, and their interaction term were entered as predictors. Age, sex, IQ, and FD were included as covariates. The omnibus cluster × group interaction was tested to determine whether the relationship between the abnormal brain region and clinical symptom score differed among groups. For analyses involving SRS‐1 measures, participants with missing SRS data were excluded using listwise deletion. Therefore, interaction analyses involving social functioning measures were conducted in participants with available SRS‐1 assessments. *p* values were corrected for multiple comparisons using the Benjamini–Hochberg false discovery rate (FDR) procedure within each significant cluster and symptom domain.

## 3 Results

### 3.1 Demographic Information

After excluding participants with excessive head motion, 53 ADHD+SD subjects, 71 ADHD–SD subjects and 72 TDC subjects were included in this analysis (see Table 1). There were no statistically significant differences among the participants in the ADHD+SD, ADHD–SD, and TDC groups in terms of age (*p* > 0.05). Moreover, there were no differences between the ADHD+SD and ADHD–SD subjects in ADHD‐RS‐IV scores. However, the three groups did differ in gender (*χ* ^2^ = 10.188, *p* = 0.006) and IQ (*F* = 25.353, *p* < 0.001); the percentage of females and the IQ in the TDC group were higher than those in the ADHD+SD and ADHD–SD group, but there were no statistically significant differences between the ADHD+SD and ADHD–SD subjects.

**TABLE 1:** Demographic data in the ADHD+SD, ADHD–SD and TDC groups.

	ADHD+SD (*n* = 53)	ADHD–SD (*n* = 71)	TDC (*n* = 72)	*F*/*χ* ^2^ stat	*p*	Post hoc
Age (months) (mean ± SD)	121.78 ± 25.92	119.64 ± 25.71	125.08 ± 24.06	0.85	0.43	
Gender (male/female)	39/14	61/10	45/27	10.19	0.01	① = ② > ③^a^
Full‐scale IQ scores	102.94 ± 10.62	105.32 ± 11.17	118.54 ± 17.29	25.35	< 0.001	① = ② < ③
ADHD‐RS‐IV						
Total	47.77 ± 8.05	46.38 ± 8.41	25.02 ± 8.87	152.70	< 0.001	① = ② > ③
Inattention	26.94 ± 3.60	26.05 ± 4.19	13.70 ± 4.43	217.16	< 0.001	① = ② > ③
Hyperactivity	20.83 ± 5.77	20.33 ± 6.35	11.32 ± 4.96	59.49	< 0.001	① = ② > ③
SRS‐1						
Total	64.68 ± 4.12	54.06 ± 3.52	53.17 ± 6.58	102.14	< 0.001	① > ② = ③
Social awareness	61.45 ± 8.20	57.06 ± 7.71	54.33 ± 8.42	9.12	< 0.001	① > ② > ③
Social cognition	66.91 ± 7.41	57.90 ± 6.26	56.17 ± 8.24	32.78	< 0.001	① > ② = ③
Social communication	63.49 ± 5.52	51.96 ± 5.56	51.33 ± 7.65	66.30	< 0.001	① > ② = ③
Social motivation	60.49 ± 6.54	52.76 ± 6.64	54.11 ± 7.09	21.32	< 0.001	① > ② = ③
Autistic mannerisms	62.58 ± 8.06	50.25 ± 4.13	49.39 ± 5.52	78.89	< 0.001	① > ② = ③

Abbreviations: ADHD‐RS‐IV, ADHD Rating Scale‐IV; SRS‐1, Social Responsiveness Scale (1st ed.).

^a^ The percentages of males in ADHD+SD and ADHD–SD were higher than in TDC (*χ* ^2^ test, *p* = 0.01). Superscript numerals indicate group labels used in the table: ① ADHD+SD; ② ADHD–SD; ③ TDC.

Among the 72 TDC participants, 36 participants had available SRS‐1 assessments and were included in the social functioning interaction analyses, whereas the remaining 36 participants did not complete SRS‐1 assessments. We compared demographic and imaging‐related variables between these two subgroups. No significant differences were observed in age, sex distribution, or mean FD between TDC participants with and without SRS‐1 data (all *p* > 0.05). However, the subgroup with available SRS‐1 assessments had significantly higher IQ scores than those without SRS‐1 data (125.6 ± 17.3 vs. 111.5 ± 14.2, Welch's *t* = −3.78, *p* < 0.001).

### 3.2 Between‐Group Differences in Amygdala‐Based FC

**FIGURE 2:**
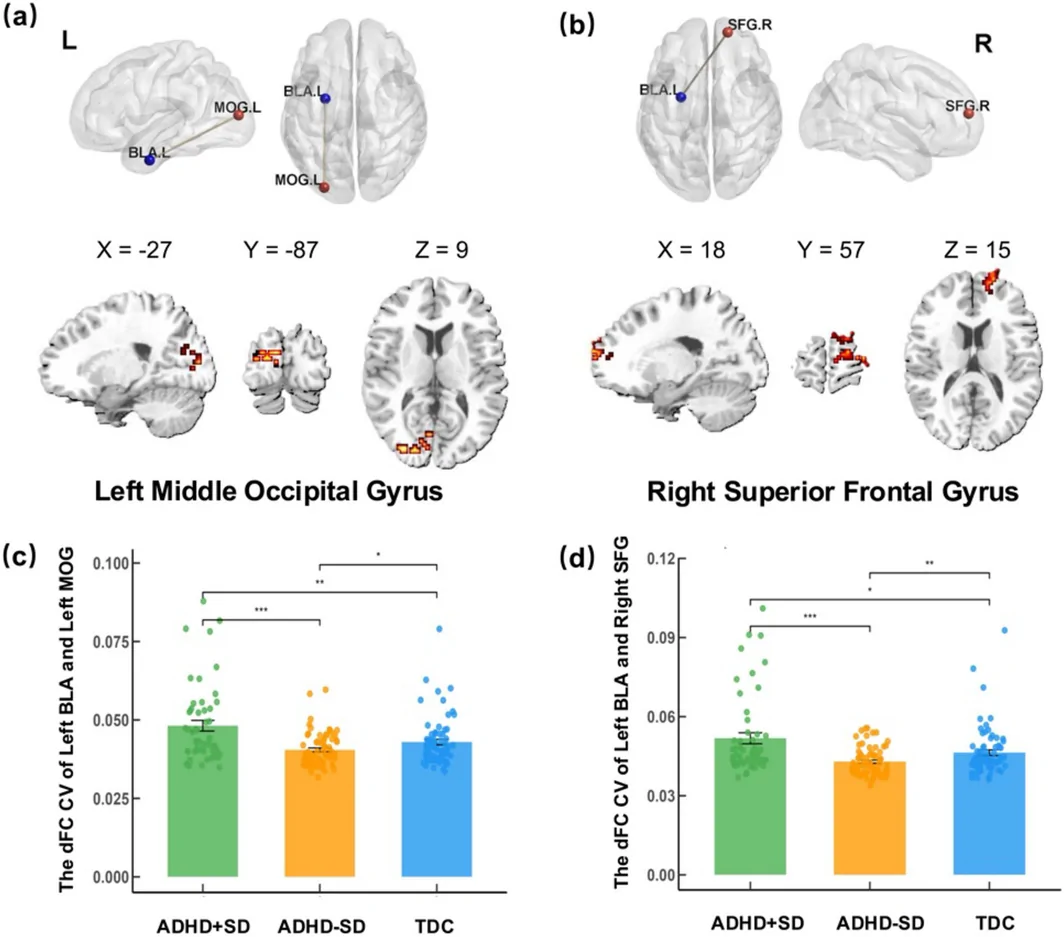
Abnormal dynamic functional connectivity (dFC) of amygdala subregions in the ADHD+SD group. (a, b) Relative to ADHD–SD and TDC, the ADHD+SD group showed significantly greater dFC variability (coefficient of variation, CV) between the left basolateral amygdala (BLA) and the left middle occipital gyrus (MOG; peak MNI −27, −87, 9; *k* = 55 voxels), and between the left BLA and the right superior frontal gyrus (SFG; peak MNI 18, 57, 15; *k* = 55 voxels). (c, d) Group comparisons of extracted dFC CV values for these two connections show a monotonic pattern (ADHD+SD > TDC > ADHD–SD). Statistics are GRF‐corrected (voxel‐wise *p* < 0.005; cluster‐level *p* < 0.05). Significance: *p < 0.05; **p < 0.01; ***p < 0.001. ADHD, attention‐deficit/hyperactivity disorder; SD, social dysfunction; BLA, basolateral amygdala; CV, coefficient of variation; dFC, dynamic functional connectivity; GRF, Gaussian random field; MNI, Montreal Neurological Institute; MOG, middle occipital gyrus; SFG, superior frontal gyrus; TDC, typically developing controls.

The sFC analysis revealed that there were no significant differences in amygdala‐based sFC among the three groups. However, the dFC analysis revealed that ADHD+SD had significantly increased CV than ADHD–SD and TDC groups between left BLA and left middle occipital gyrus (MOG) (peak MNI: −27, −87, 9; voxel size = 55), as well as between the left BLA and right superior frontal gyrus (SFG) (peak MNI: 18, 57, 15; voxel size = 55). We extracted the dFC CV values for these connections across the three groups. The results revealed a consistent pattern, with the highest CV values observed in the ADHD+SD group, followed by the TDC group, and the lowest in the ADHD–SD group. Importantly, validation analyses confirmed that these two dFC alterations were highly robust to methodological variations, remaining significant across a wide range of sliding‐window lengths (30, 40, and 60 TRs) and varying step sizes (1 and 2 TRs) (see Figure 2 and Table 2). No significant differences were observed in BLA–MOG or BLA–SFG dFC values between TDC participants with and without SRS‐1 assessments.

**TABLE 2:** Brain regions with significant dFC variability differences among children in the ADHD+SD, ADHD–SD and TDC groups.

Comparison	Seed ROI	Regions	Peak MNI coordinate	Cluster size	Peak *z*‐value
ADHD+SD vs. ADHD–SD	Left BLA	Left middle occipital gyrus	(−27, −87, 9)	55	3.93
		Right superior frontal gyrus	(18, 57, 15)	55	4.02

*Note:* Whole‐brain voxel‐wise analysis revealed significant differences in dFC variability only between ADHD+SD and ADHD–SD (GRF‐corrected: voxel‐wise *p* < 0.005; cluster‐level *p* < 0.05). Mean dFC coefficient‐of‐variation (CV) values extracted from the basolateral amygdala (BLA) showed a monotonic pattern across groups (ADHD+SD > TDC > ADHD–SD).

Abbreviations: BLA, basolateral amygdala; CV, coefficient of variation; dFC, dynamic functional connectivity; GRF, Gaussian random field; TDC, typically developing controls.

### 3.3 Group‐Dependent Associations Between Significant Clusters and Clinical Symptom Dimensions

We further examined whether the associations between dFC CV values extracted from the significant clusters and clinical symptom dimensions differed across groups. For ADHD‐RS‐IV symptom dimensions, no cluster × group interaction survived FDR correction. For SRS‐1 measures, a significant left BLA and left MOG × group interaction was observed for social motivation, *F*(2, 150) = 5.07, *p* = 0.007, FDR *q* = 0.045, indicating that the association between left BLA and left MOG dFC variability and social motivation differed across groups. Simple slope analyses showed that higher left BLA and left MOG dFC variability was associated with greater social motivation impairment in ADHD–SD group (*β* = 368.81, *p* = 0.021), whereas the association was negative in TDC group (*β* = −457.86, *p* = 0.036) and not evident in ADHD+SD group (*β* = −79.60, *p* = 0.411). No significant group‐dependent association was observed for left BLA and right SFG.

## 4 Discussion

To our knowledge, this is the first study to contrast amygdala and whole‐brain FC patterns in ADHD individuals with and without SD. We found no significant ADHD+SD‐specific alterations in static amygdala connectivity, whereas dFC analyses revealed increased temporal variability between the left BLA and both the left MOG and right SFG in the ADHD+SD group. Symptom interaction analyses further showed a group‐dependent association between BLA–MOG dFC variability and social motivation. These findings suggest that social dysfunction in ADHD may be more closely related to altered temporal coordination of amygdala–cortical interactions than to changes in average static connectivity.

Contrary to our hypothesis, ADHD+SD did not exhibit significant abnormalities in static amygdala FC. Previous resting‐state fMRI studies have reported altered amygdala FC in ADHD, particularly involving the prefrontal, temporal, insular cortices [41, 42], although the direction and spatial distribution of these abnormalities have varied considerably across studies. Some studies reported reduced amygdala‐prefrontal connectivity associated with impaired emotional regulation and social cognition, whereas others observed increased connectivity that was interpreted as reflecting delayed neurodevelopment or compensatory mechanisms [41, 43, 44]. Similar heterogeneity has also been reported in ASD‐related studies [45, 46].

The discrepancy between previous findings and the present study may be explained by several factors. First, most previous studies compared ADHD participants with TDCs, whereas the present study specifically contrasted ADHD children with and without SD, thereby focusing on the neural substrates underlying SD rather than ADHD diagnosis itself. Second, differences in participant characteristics, including age, symptom severity, medication status, amygdala subregion definition, preprocessing strategies, and analytical approaches, may also contribute to the inconsistent findings across studies [41, 42]. Therefore, the absence of significant sFC differences in the present study does not necessarily indicate preserved amygdala function, but rather suggests that SD in ADHD may be more closely associated with alterations in the temporal organization of functional interactions than with alterations in average connectivity. The present findings therefore extend previous evidence of amygdala dysregulation by suggesting that amygdala dysfunction may be expressed not only as altered context‐dependent functional coupling during externally driven social–emotional processing but also as disrupted temporal organization of intrinsic amygdala‐centered networks at rest.

Although sFC reflects the brain's ability to maintain stable and constant connectivity patterns over extended timescales, dFC captures the variability and stability of integrating, coordinating, and reconfiguration of functional interactions [19, 20, 47]. Social interaction depends on rapid neural adaptation, with circuits reorganizing as cues are perceived, evaluated, and acted upon [48, 49]. When connectivity is averaged across the entire scan, these brief but behaviorally relevant adjustments are compressed into a single value; preserved sFC in ADHD+SD therefore may reflect the absence of detectable alterations in average connectivity rather than intact moment‐to‐moment coordination [28, 50]. By contrast, the robust dFC effects indicate abnormalities in the timing and configuration of amygdala‐cortical coupling. Greater temporal instability may reflect less reliable integration between amygdala‐centered socio‐emotional systems and distributed visual and cognitive control networks [20, 51]. Thus, the dissociation of null sFC and abnormal dFC implies that ADHD+SD is defined not by altered average connection strength but by disrupted temporal coordination of otherwise relatively typical networks [48, 51].

Recent task‐based fMRI studies have reported altered amygdala functional connectivity during social–emotional processing in children with ADHD, suggesting abnormal context‐dependent recruitment of amygdala‐centered circuits [52]. Rather than contradicting these findings, the absence of resting‐state sFC abnormalities in the present study likely reflects the distinct aspects of brain function captured by task‐based, resting‐state static, and resting‐state dFC. Conceptually, task‐based FC primarily reflects context‐dependent recruitment of functional circuits, resting‐state sFC characterizes their average intrinsic coupling, whereas resting‐state dFC captures the temporal organization and variability of intrinsic functional interactions. Our findings therefore suggest that amygdala dysfunction in ADHD+SD is expressed primarily as disrupted temporal coordination at rest, complementing previous task‐based evidence of abnormal functional recruitment during social–emotional processing.

In the dFC analysis, we found that in the ADHD+SD group, the brain regions showing increased FC instability were primarily located along BLA‐centered visual‐perceptual and higher‐order cognitive control pathways. The BLA receives sensory information and assigns affective salience to relevant stimuli before transmitting this information to downstream cortical systems [53, 54, 55]. Therefore, altered BLA‐centered temporal coupling may affect the integration of affective salience with perceptual and regulatory processes that are important for social functioning [56].

The most clinically informative effect involved the BLA–MOG connection. As part of the visual cortex, the MOG is involved in visual information processing and integration [57]. Increased BLA–MOG dFC variability in ADHD+SD may reflect less stable temporal coordination between affective salience processing and visually mediated social cue processing [58, 59]. Importantly, the interaction analysis showed that the relationship between BLA–MOG dFC variability and social motivation differed across groups. Post hoc slope estimates showed that BLA–MOG variability positively tracked social motivation impairment in ADHD–SD, whereas the association was negative in TDC and not clearly evident in ADHD+SD. This pattern suggests that BLA–MOG variability may have different behavioral meanings depending on social dysfunction status. In ADHD children without marked social dysfunction, BLA–MOG variability may still index individual differences in subclinical social motivation difficulties. In ADHD+SD, however, BLA–MOG variability was already elevated at the group level and no longer showed a linear relationship with social motivation, suggesting that this circuit‐level instability may reflect a more established alteration in affective‐visual coordination. This interpretation remains exploratory and requires replication in independent samples.

In parallel, ADHD+SD also showed increased instability in BLA–SFG connectivity. The SFG plays a crucial role in higher‐order cognitive functions, particularly executive control, attention, and working memory [60, 61]. Previous studies have reported structural and functional abnormalities of the SFG in ADHD, and SFG alterations have been linked to core ADHD symptoms and cognitive regulation [62, 63, 64]. Increased BLA–SFG dFC variability may therefore reflect altered temporal coordination between affective salience systems and frontal control regions. Unlike the BLA–MOG connection, however, BLA–SFG variability did not show an FDR‐corrected group‐dependent association with SRS‐derived symptom dimensions. Thus, its clinical interpretation should be more conservative. Rather than serving as a symptom‐specific marker of social motivation, BLA–SFG instability may reflect a broader alteration in amygdala‐frontal coordination in ADHD+SD. Future studies using task‐based social cognition and emotion‐regulation paradigms are needed to determine how affective‐visual and affective‐control pathways jointly contribute to social dysfunction in ADHD.

However, this study has some limitations. First, the amygdala is involved in multiple aspects of emotion and social behavior, and its functional complexity may make interpreting results challenging. Second, sliding‐window dFC involves several user‐defined parameters and has relatively limited temporal resolution. Although we further performed sensitivity analyses using different window lengths (30, 40 and 60 TRs) and step sizes (2 TRs), obtaining highly consistent results, sliding‐window‐based dFC remains inherently dependent on parameter selection. The measurement methods and parameter choices for dFC may affect the stability of the results. Additionally, this study conducted analyses only at the voxel level. Future research should extend to the regional level (e.g., AAL 90) and the network level (e.g., Yeo 7 networks).

Finally, although SRS‐1 assessments were available for only half of the TDC participants, comparisons between TDC participants with and without SRS‐1 data showed no significant differences in age, sex, head motion, or BLA–MOG and BLA–SFG dFC values. However, participants with available SRS‐1 assessments had significantly higher IQ scores than those without SRS‐1 data. Importantly, IQ was included as a covariate in all brain–behavior interaction analyses, together with age, sex, and mean FD, thereby reducing its potential influence on the reported interaction effects. Nevertheless, because participants with available SRS‐1 assessments may represent a cognitively higher‐functioning subset, potential selection bias related to cognitive ability cannot be completely excluded. Therefore, the present interaction findings should be interpreted with appropriate caution until replicated in larger samples with complete behavioral assessments. Future research should include larger‐scale samples and employ methods with higher temporal resolution, such as coactivation approaches, to further analyze the dynamic connectivity features of the amygdala in ADHD and their relationship with social function.

## 5 Conclusion

In conclusion, our findings demonstrate that SD in childhood ADHD is closely linked to increased temporal instability in amygdala connectivity. The heightened dFC variability in the ADHD+SD group suggests that disrupted neural dynamics represent a critical mechanism of SD, extending our understanding beyond traditional static network models. Specifically, the group‐dependent association between BLA–MOG dynamic variability and social motivation suggests that altered amygdala‐visual coordination may drive individual differences in social functioning. These results emphasize the necessity of integrating dynamic connectivity approaches to untangle the complex neurobiology of ADHD, paving the way for more precise, network‐level diagnostic and interventional strategies.

## Author Contributions

K.Z. conceived and designed the experiment. K.Z., X.P., J.Y., Y.Z. and Z.F. collected the data. K.Z. performed the data analyses. H.Y. and Y.X. supervised the data analyses. Z.C. and Q.C. revised the manuscript. All authors contributed to the discussion of the manuscript. All authors read and approved the final manuscript.

## Funding

This work was supported by grants from the Brain Science and Brain‐like Intelligence Technology—National Science and Technology Major Project (2021ZD0200500), and the National Natural Science Foundation of China (82371548).

## Ethics Statement

This study was conducted under the approval of the Ethics Committee of Peking University Sixth Hospital (Approval No. 3013‐Youth‐3). This article does not contain any studies with animals performed by any of the authors. The participants and their parents were asked to sign an informed consent prior to their participation in the study.

## Consent

All authors agreed the possible publication of this article on Child and Adolescent Psychiatry and Mental Health. The participant has consented to the submission of the article to the journal.

## Conflicts of Interest

The authors declare no conflicts of interest.

## Data Availability

The data that support the findings of this study are available from the corresponding author upon reasonable request.
